# Supplementary material for: Absolute excited state molecular geometries revealed by resonance Raman signals
Source: Nat Commun. 2022 Dec 15;13:7770. doi: 10.1038/s41467-022-35099-3 (PMC9755279; doi:10.1038/s41467-022-35099-3)
Supplement: Supplementary file 1 — Supplementary Information [file 41467_2022_35099_MOESM1_ESM.pdf]

Supplementary Information
Absolute excited state molecular geometries revealed by resonance Raman signals

Giovanni Batignani[1,2,†,*], Emanuele Mai[1,2,†], Giuseppe Fumero[1], Shaul Mukamel[3], Tullio Scopigno[1,2,4,*]
[1]*Dipartimento di Fisica, Sapienza Università di Roma, P.le Aldo Moro 5, Roma I-00185, Italy*
[2]*Istituto Italiano di Tecnologia, Center for Life Nano Science @Sapienza, Viale Regina Elena 291, Roma I-00161, Italy*
[3]*Department of Chemistry, University of California, Irvine, 92623, California, USA*
[4]*Istituto Italiano di Tecnologia, Graphene Labs, Via Morego 30, Genova I-16163, Italy*

[†]*These authors contributed equally to this work*
[*]*giovanni.batignani@uniroma1.it*
[*]*tullio.scopigno@uniroma1.it*

SUPPLEMENTARY NOTE 1: COMPLETE DERIVATION OF THE ISRS SIGNAL.

The different contributions concurring to the ISRS signal can be computed via the Feynman diagrams introduced in Fig.1 of the Main Text. Specifically, they take into account for two classes of processes, depending on whether the two interactions with the off-resonant Raman pump (RP) involve the ket (A, Fig.1(a)) or the bra-side of the density matrix (B, Fig.1(b)). Among each class, the two interactions with the probe pulse (PP) project the system onto the entire electronic excited-state (ES) manifold (with a transition amplitude ruled, according to the Franck-Condon principle [1], by the overlap between the ground and excited states vibrational eigenfunctions).

Importantly, the off-resonant RP ensures the generation of vibrational coherences only on the initially populated electronic level (specifically, $|g'_j\rangle\langle g_j|$ for the A diagrams and $|g_j\rangle\langle g'_j|$ for the B ones), modulating the optical properties of the system at the frequencies Ω_j of the simulated normal modes (j). Hereafter we focus, without loss of generality, on a specific ground state (GS) mode, defining $\omega_{g'g} = \Omega_j$ and dropping the subscript j. Hence, the total ISRS signal related to such a mode is given by the sum over all the levels $|e_k\rangle$ belonging to the ES vibrational manifold, including all the possible transitions among the different normal modes.

Starting from the A diagrams (Fig.1(a)), the third order polarization which generates the signal is [2]

$$P_A^{(3)}(\omega) = \sum_{e_k}\sum_{\epsilon}\left(\frac{i}{\hbar}\right)^3 \int_{-\infty}^{+\infty} dt\, e^{i\omega t}\mu_{R_1}\mu_{R_2}^*\,\mu_{g'\,e_k}\mu_{e_k\,g}\int_{-\infty}^{t} dt_3\int_{-\infty}^{t_3} dt_2\int_{-\infty}^{t_2} dt_1$$
$$E_R(t_1)E_R^*(t_2)E_P(t_3)e^{-i\tilde{\omega}_{\epsilon g}(t_2-t_1)}e^{-i\tilde{\omega}_{g'g}(t_3-t_2)}e^{-i\tilde{\omega}_{e_k g}(t-t_3)} \quad \text{(S1)}$$

where $\tilde{\omega}_{ij} = \omega_i - \omega_j - i\Gamma_{ij}$ and $\mu_{R_1}\mu_{R_2}^*$ are the dipole elements of the transitions induced by the RP. Being the latter off-resonant, the related summation runs over all the energy levels ϵ of the molecule.

When the RP and the PP are not overlapped in time, the integral over t_2 can be extended from $-\infty$ to $+\infty$, taking advantage of the absence of the field temporal overlap to cancel the contributions for $t_2 > t_3$ [3]:

$$P_A^{(3)}(\omega) = \sum_{\epsilon,\,e_k}\left(\frac{i}{\hbar}\right)^3 \int_{-\infty}^{+\infty} dt\, e^{i\omega t}\mu_{R_1}\mu_{R_2}^*\,\mu_{g'\,e_k}\mu_{e_k\,g}\int_{-\infty}^{t} dt_3\int_{-\infty}^{\infty} dt_2\int_{-\infty}^{t_2} dt_1$$
$$E_R(t_1)E_R^*(t_2)E_P(t_3)e^{-i\tilde{\omega}_{\epsilon g}(t_2-t_1)}e^{-i\tilde{\omega}_{g'g}(t_3-t_2)}e^{-i\tilde{\omega}_{e_k g}(t-t_3)} \quad \text{(S2)}$$

The two contributions related to the interaction with the RP and with the PP can be hence treated separately, recasting Eq.S2 as

$$P_A^{(3)}(\omega) = A_R \cdot A_P \quad \text{(S3)}$$

with

$$A_R = \sum_{\epsilon}\mu_{R_1}\mu_{R_2}^*\int_{-\infty}^{\infty} dt_2\int_{-\infty}^{t2} dt_1\, E_R(t_1)E_R^*(t_2)e^{-i\tilde{\omega}_{\epsilon g}(t_2-t_1)}e^{+i\tilde{\omega}_{g'g}(t_2)}$$

and

$$A_P = \sum_{e_k}\mu_{g'\,e_k}\mu_{e_k\,g}\left(\frac{i}{\hbar}\right)^3\int_{-\infty}^{+\infty} dt\, e^{i\omega t}\int_{-\infty}^{t} dt_3\, E_P(t_3)e^{-i\tilde{\omega}_{g'g}(t_3)}e^{-i\tilde{\omega}_{e_k g}(t-t_3)}$$

The A_R term, taking into account for the RP vibrational preparation, can be rewritten, via the change of variables $t_1 = \tau - s$ and $t_2 = \tau + s$, as

$$A_R = \sum_\epsilon \mu_{R_1}\mu^*_{R_2} \int_{-\infty}^{\infty} d\tau \int_0^\infty ds\, E_R(\tau - s)E^*_R(\tau + s)e^{-2i\tilde{\omega}_{\epsilon g}(s)}e^{+i\tilde{\omega}_{g'g}(\tau+s)}$$

Expanding the fields in terms of their Fourier components as $E_R(t) = \int \frac{d\omega'}{2\pi} E_R(\omega')e^{-i\omega' t}$, we obtain

$$A_R = \sum_\epsilon \mu_{R1}\mu^*_{R2} \int \frac{d\omega'}{2\pi} E_R(\omega') \int \frac{d\omega''}{2\pi} E^*_R(\omega'') \int_{-\infty}^{\infty} d\tau\, e^{i\tau(\omega''-\omega'+\tilde{\omega}_{g'g})} \int_0^\infty ds\, e^{is(\omega''+\omega'-2\tilde{\omega}_{\epsilon g}+\tilde{\omega}_{g'g})}$$

where it holds

$$\int_0^\infty ds\, e^{is(\omega''+\omega'-2\tilde{\omega}_{\epsilon g}+\tilde{\omega}_{g'g})} = \frac{-1}{i(\omega'' + \omega' - 2\tilde{\omega}_{\epsilon g} + \tilde{\omega}_{g'g})}$$

and, for a small vibrational damping $\Gamma_{g'g} \approx 0$:

$$\int_{-\infty}^{\infty} d\tau\, e^{i\tau(\omega''-\omega'+\tilde{\omega}_{g'g})} = 2\pi\delta(\omega'' - \omega' + \omega_{g'g})$$

Hence,

$$A_R = \sum_\epsilon \mu_{R_1}\mu^*_{R_2} \int \frac{d\omega'}{2\pi} \frac{-E_R(\omega')E^*_R(\omega' - \omega_{g'g})}{2i(\omega' - \tilde{\omega}_{\epsilon g})} \tag{S4}$$

The PP response A_P in the expression of $P^{(3)}_A(\omega)$ can be rewritten as

$$A_P = \sum_{e_k} \mu_{g'\,e_k}\mu_{e_k\,g} \left(\frac{i}{\hbar}\right)^3 \int_{-\infty}^{+\infty} \frac{d\omega_P}{2\pi} E_P(\omega_P) \int_{-\infty}^{+\infty} dt\, e^{i(\omega-\tilde{\omega}_{e_k g})t} \int_{-\infty}^{t} dt_3\, e^{-i(\omega_P+\tilde{\omega}_{g'g}-\tilde{\omega}_{e_k g})t_3} =$$

$$= \sum_{e_k} \mu_{g'\,e_k}\mu_{e_k\,g} \left(\frac{i}{\hbar}\right)^3 \int_{-\infty}^{+\infty} \frac{d\omega_P}{2\pi} E_P(\omega_P) \int_{-\infty}^{+\infty} dt\, \frac{e^{i(\omega-\omega_P-\tilde{\omega}_{g'g})t}}{-i(\omega_P + \tilde{\omega}_{g'g} - \tilde{\omega}_{e_k g})}$$

which, for a small vibrational damping $\Gamma_{g'g} \approx 0$, can be recast as

$$A_P = \sum_{e_k} \mu_{g'\,e_k}\mu_{e_k\,g} \left(\frac{i}{\hbar}\right)^3 \int_{-\infty}^{+\infty} d\omega_P\, E_P(\omega_P) \frac{\delta(\omega - \omega_P - \omega_{g'g})}{-i(\omega_P + \omega_{g'g} - \tilde{\omega}_{e_k g})} = \sum_{e_k} \mu_{g'\,e_k}\mu_{e_k\,g} \left(\frac{i}{\hbar}\right)^3 \frac{E_P(\omega - \omega_{g'g})}{-i(\omega - \tilde{\omega}_{e_k g})} \tag{S5}$$

Hence, Eq.s S3, S4, S5 yield

$$P^{(3)}_A(\omega) = \sum_{e_k} \frac{i}{\hbar^3} \mu_{g'\,e_k}\mu_{e_k\,g} \frac{E_P(\omega - \omega_{g'g})}{(\omega - \tilde{\omega}_{e_k g})} \sum_\epsilon \mu_{R_1}\mu^*_{R_2} \int \frac{d\omega'}{2\pi} \frac{E_R(\omega')E^*_R(\omega' - \omega_{g'g})}{2(\omega' - \tilde{\omega}_{\epsilon g})} \tag{S6}$$

Considering a RP centered at $t = -T$ and a PP centered at $t = 0$, it holds

$$E_R = E_R(t + T) \Rightarrow E_R(\omega) = \int_{-\infty}^{+\infty} dt\, E_R(t + T)e^{i\omega t} = E^0_R(\omega)\, e^{-i\omega T}$$

where $E^0_R(\omega)$ is the Fourier Transform of the pulse temporal envelope centered at $t = 0$. Eq. S6 can hence be rewritten as

$$P^{(3)}_A(\omega) = \sum_{e_k} \frac{i}{\hbar^3} \mu_{g'\,e_k}\mu_{e_k\,g}\, e^{-i\omega_{g'g}T} \frac{E_P(\omega - \omega_{g'g})}{(\omega - \tilde{\omega}_{e_k g})} \sum_\epsilon \mu_{R_1}\mu^*_{R_2} \int \frac{d\omega'}{2\pi} \frac{E^0_R(\omega')E^{0*}_R(\omega' - \omega_{g'g})}{2(\omega' - \tilde{\omega}_{\epsilon g})}$$

Notably, for an off-resonant RP the denominator of the integrated function is real, as well as the numerator for a (nearly) transform limited RP. Thus, the second summation yields a real constant, which is the vibrational preparation (VP) for the A processes:

$$VP^A_{g'} = -\sum_\epsilon \mu_{R_1}\mu^*_{R_2} \int \frac{d\omega'}{2\pi} \frac{E^0_R(\omega')E^{0*}_R(\omega' - \omega_{g'g})}{2(\omega' - \tilde{\omega}_{\epsilon g})} \tag{S7}$$

This relation can be exploited for expressing the nonlinear polarization $P_A^{(3)}(\omega,T)$ as

$$P_A^{(3)}(\omega,T) = -VP_{g'}^A \sum_{e_k} \frac{i}{\hbar^3}\,\mu_{g'\,e_k}\mu_{e_k\,g}\,e^{-i\omega_{g'g}T}\,\frac{E_P(\omega-\omega_{g'g})}{(\omega-\tilde{\omega}_{e_k g})} \tag{S8}$$

Finally, the response related to the A processes can be obtained via Eq.(1) of the Main Text as

$$\boxed{S_A(\omega,T) = VP_{g'}^A\;\Re\left[e^{-i\omega_{g'g}T}\,\frac{E_P(\omega-\omega_{g'g})}{E_P(\omega)}\sum_{e_k}\frac{\mu_{g'\,e_k}\mu_{e_k\,g}}{\omega-\tilde{\omega}_{e_k g}}\right]} \tag{S9}$$

The contribution of the diagrams in Fig.1(b) to the measured ISRS signal can be computed by following a procedure analogue to the one described above. The final expression of the response related to the B processes reads as

$$\boxed{S_B(\omega,T) = -VP_{g'}^B\;\Re\left[e^{-i\omega_{gg'}T}\,\frac{E_P(\omega-\omega_{gg'})}{E_P(\omega)}\sum_{e_k}\frac{\mu_{ge_k}\mu_{e_k g'}}{\omega-\tilde{\omega}_{e_k g'}}\right]} \tag{S10}$$

where

$$VP_{g'}^B = -\sum_{\epsilon}\mu_{R_1}^*\mu_{R_2}\int\frac{d\omega'}{2\pi}\,\frac{E_R^0(\omega')E_R^{0*}(\omega'-\omega_{gg'})}{2(\omega'+\tilde{\omega}_{g\epsilon}-\omega_{gg'})}$$

In the following we show that, for an off-resonant RP, the vibrational preparation response for the A and B processes are the same. Indeed, recalling the definition $\tilde{\omega}_{ij} = \omega_i - \omega_j - i\Gamma_{ij}$, $VP_{g'}^B$ can be rewritten as

$$VP_{g'}^B = -\sum_{\epsilon}\mu_{R_1}^*\mu_{R_2}\int\frac{d\omega'}{2\pi}\,\frac{E_R^0(\omega')E_R^{0*}(\omega'+\omega_{g'g})}{2(\omega'-\omega_{\epsilon g}+\omega_{g'g}-i\Gamma_{\epsilon g})} = -\sum_{\epsilon}\mu_{R_1}^*\mu_{R_2}\int\frac{d\omega''}{2\pi}\,\frac{E_R^0(\omega''-\omega_{g'g})E_R^{0*}(\omega'')}{2(\omega''-\omega_{\epsilon g}-i\Gamma_{\epsilon g})} \tag{S11}$$

where the equality $\Gamma_{g\epsilon} = \Gamma_{\epsilon g}$ has been exploited and, in the last step, the change of variables $\omega'' = \omega' + \omega_{g'g}$ has been performed.

Hence, by comparison with Eq.(S7), in general

$$VP_{g'}^B = (VP_{g'}^A)^*$$

Notably, in the harmonic approximation the dipole matrix elements μ_{R_1}, μ_{R_2} are real, as well as $E_R^0(\omega)$ for a (nearly) transform-limited RP. Moreover, being the RP off-resonant, it holds $|\omega''-\omega_{\epsilon g}| \gg \Gamma_{\epsilon g}$, so that both the vibrational preparation responses are real. Thus, for the chosen experimental configuration

$$VP_{g'}^B = VP_{g'}^A$$

The obtained Eqs. S7 and S11 can be similarly expressed in terms of the molecular polarizability $\alpha(t)$, by evaluating the vibrational coherences preparation via the off-resonant Raman pump through the interaction Hamiltonian[4]

$$H_I^{(RP)} = -\alpha(t)\,|E_R(t)|^2$$

Expanding the $\alpha(t)$ in terms of the normal coordinates Q_j allows for evaluating the preparation function between the vibrational coherences ($\langle g_j'|\ldots|g\rangle$) under consideration, leading to the expression for $VP_{g'}^{RP}$ reported in Eq.3 of the Main Text.

The total ISRS signal related to a specific normal mode is then obtained from Eqs. S9-S10 as

$$S(\omega,T) \propto \operatorname{sgn}\Big(\frac{\partial\alpha}{\partial Q_{g'}}\Big)\cdot$$
$$\Re\left[e^{-i\omega_{g'g}T}\,\frac{E_P(\omega-\omega_{g'g})}{E_P(\omega)}\sum_{e_k}\frac{\mu_{g'\,e_k}\mu_{e_k\,g}}{\omega-\tilde{\omega}_{e_k g}} - e^{-i\omega_{gg'}T}\,\frac{E_P(\omega+\omega_{g'g})}{E_P(\omega)}\sum_{e_k}\frac{\mu_{ge_k}\mu_{e_k g'}}{\omega-\tilde{\omega}_{e_k g'}}\right]$$

and, finally, Eqs.5 of the Main Text are retrieved[1] by taking advantage of the complex REPs definitions in Eq.4, namely $R_{g'}^S(\omega)$ and $R_{g'}^{AS}(\omega)$ (related to the Stokes and anti-Stokes processes, respectively). These latter will also be used hereinafter.

---

[1] for notation clarity, in the Main Text the RP-PP delay has been denoted with ΔT instead of T

It is worth to stress that we avoided referring to stimulated Raman pathways A/B as either Stokes or anti-Stokes, since such a classification can be ambiguous. In fact, according to the most common nomenclature, the distinction between Stokes and anti-Stokes processes is based on the temporally last Raman interaction. One speaks of a Stokes process when, due to the last interaction, the molecule passes from a state of lower to higher energy and the emission is red-shifted; conversely in anti-Stokes the last transition is from a state of higher energy to one of lower energy and the emission is blue-shifted (the term coherent anti-Stokes Raman spectroscopy comes from this convention). One can alternatively distinguish Stokes and anti-Stokes processes by looking at the overall energy transfer between the matter and the fields, during the entire process with all interactions, not just the last. Stokes processes start from the ground state, while anti-Stokes processes start from vibrationally excited states. Another alternative is to distinguish Stokes and anti-Stokes processes by looking at the dependence of the Raman response to the probed wavelength: in the impulsive Raman case, due to the excitation of the vibrational coherence induced by a pump field different with respect to the probe, Feynman pathways are sensitive to either Stokes or anti-Stokes resonant Raman excitation profiles of spontaneous Raman spectroscopy (see Eqs. 2, 4 of the Main text).

Dissecting the complex Raman excitation profiles tuning the experimental parameters.

For a chirped probe pulse the $E_P(\omega)$ field can be expressed as

$$E_P(\omega) = E_P^0(\omega) e^{iC_2(\omega-\omega_P)^2}$$

where $E_P^0(\omega) = \sqrt{I_P(\omega)}$ is a real quantity, C_2 is the probe chirp and ω_P is the spectral component of the PP arriving at $T = 0$.
Substituting this relation in Eq.S9, the A signal $S_A(\omega, t)$ can be expressed as

$$S_A(\omega, T) \propto \mathrm{sgn}\Big(\frac{\partial\alpha}{\partial Q_{g'}}\Big)\,\Re\left[\frac{E_P^0(\omega-\omega_{g'g})}{E_P^0(\omega)}\, e^{-i\omega_{g'g}T}\, e^{iC_2(\omega-\omega_P-\omega_{g'g})^2}\, e^{-iC_2(\omega-\omega_P)^2}\, R_{g'}^S(\omega)\right] =$$

$$\mathrm{sgn}\Big(\frac{\partial\alpha}{\partial Q_{g'}}\Big)\,\frac{E_P^0(\omega-\omega_{g'g})}{E_P^0(\omega)}\,\Re\left[e^{-i\omega_{g'g}T} e^{-2iC_2(\omega-\omega_P)\omega_{g'g}+iC_2\omega_{g'g}^2}\, R_{g'}^S(\omega)\right]$$

Similarly, for the B terms, Eq.S10 can be recast as

$$S_B(\omega, T) \propto -\mathrm{sgn}\Big(\frac{\partial\alpha}{\partial Q_{g'}}\Big)\,\Re\left[\frac{E_P^0(\omega-\omega_{gg'})}{E_P^0(\omega)}\, e^{-i\omega_{gg'}T}\, e^{iC_2(\omega-\omega_P-\omega_{gg'})^2}\, e^{-iC_2(\omega-\omega_P)^2}\, R_{g'}^{AS}(\omega)\right] =$$

$$-\mathrm{sgn}\Big(\frac{\partial\alpha}{\partial Q_{g'}}\Big)\,\frac{E_P^0(\omega+\omega_{g'g})}{E_P^0(\omega)}\,\Re\left[e^{i\omega_{g'g}T}\, e^{2iC_2(\omega-\omega_P)\omega_{g'g}+iC_2\omega_{g'g}^2}\, R_{g'}^{AS}(\omega)\right]$$

For convenience, we can define the phase term

$$\boxed{\phi = \phi(\omega, T, C_2) = \omega_{g'g}T + 2\,C_2(\omega-\omega_P)\,\omega_{g'g} = \tilde{T}(\omega, C_2)\,\omega_{g'g}}$$

which is the product between the arrival time of the monitored probe wavelength, i.e. $\tilde{T}(\omega, C_2) = T + 2\,C_2(\omega-\omega_P)$, and the frequency of the considered normal mode $\omega_{g'g}$.
For a flat PP spectral profile in the region of interest ($E_P^0(\omega \pm \omega_{g'g}) \approx E_P^0(\omega)$),

$$\begin{cases} S_A(\omega, T) \propto \mathrm{sgn}\Big(\frac{\partial\alpha}{\partial Q_{g'}}\Big)\,\Re\left[e^{-i\phi}\, e^{iC_2\omega_{g'g}^2}\, R_{g'}^S(\omega)\right] \\ S_B(\omega, T) \propto -\mathrm{sgn}\Big(\frac{\partial\alpha}{\partial Q_{g'}}\Big)\,\Re\left[e^{+i\phi}\, e^{iC_2\omega_{g'g}^2}\, R_{g'}^{AS}(\omega)\right] \end{cases}$$

so that the ISRS signal of the g' mode reads

$$\begin{aligned} \frac{S(\omega, T)}{\mathrm{sgn}\Big(\frac{\partial\alpha}{\partial Q_{g'}}\Big)} &\propto \Re\left\{e^{iC_2\,\omega_{g'g}^2}\,\left[e^{-i\phi}R_{g'}^S(\omega) - e^{+i\phi}R_{g'}^{AS}(\omega)\right]\right\} = \\ &\cos\left(C_2\,\omega_{g'g}^2\right)\Re\left[e^{-i\phi}R_{g'}^S(\omega) - e^{+i\phi}R_{g'}^{AS}(\omega)\right] \\ &-\sin\left(C_2\,\omega_{g'g}^2\right)\Im\left[e^{-i\phi}R_{g'}^S(\omega) - e^{+i\phi}R_{g'}^{AS}(\omega)\right] \end{aligned} \tag{S12}$$

Hence, the real/imaginary part of the term $\left[e^{-i\phi}\,R^{S}_{g'}(\omega)\,-e^{+i\phi}\,R^{AS}_{g'}(\omega)\right]$ can be measured, in a selective manner, via a proper tuning of the probe chirp C_2. Particularly, as shown in the following, through selected values of the time delay T it is possible to measure independently the complex REPs $R^{S}_{g'}(\omega)$ and $R^{AS}_{g'}(\omega)$.
Indeed, the first term in Eq.S12 can be rewritten as

$$\Re\left[e^{-i\phi}R^{S}_{g'}(\omega)-e^{+i\phi}R^{AS}_{g'}(\omega)\right]=$$
$$=\Re\Big[\left[\cos(\phi)-i\sin(\phi)\right]R^{S}_{g'}(\omega)-\left[\cos(\phi)+i\sin(\phi)\right]R^{AS}_{g'}(\omega)\Big]=$$
$$=\cos(\phi)\,\Re\left[R^{S}_{g'}(\omega)-R^{AS}_{g'}(\omega)\right]+\sin(\phi)\,\Im\left[R^{S}_{g'}(\omega)+R^{AS}_{g'}(\omega)\right]$$

while the second as

$$\Im\left[e^{-i\phi}R^{S}_{g'}(\omega)-e^{+i\phi}R^{AS}_{g'}(\omega)\right]=-\sin(\phi)\,\Re\left[R^{S}_{g'}(\omega)+R^{AS}_{g'}(\omega)\right]+\cos(\phi)\,\Im\left[R^{S}_{g'}(\omega)-R^{AS}_{g'}(\omega)\right]$$

Hence, the ISRS signal of the considered mode in Eq.S12 can be expressed as in Eq.6 of the Main Text, which is rewritten in the following for convenience:

$$\boxed{\begin{gathered}S(\omega,T,C_2)\propto\operatorname{sgn}\Big(\frac{\partial\alpha}{\partial Q_{g'}}\Big)\cdot\\ \Big[\cos\left(C_2\,\omega^2_{g'g}\right)\Big\{\cos(\phi)\,\Re\left[R^{S}_{g'}(\omega)-R^{AS}_{g'}(\omega)\right]+\sin(\phi)\,\Im\left[R^{S}_{g'}(\omega)+R^{AS}_{g'}(\omega)\right]\Big\}+\\ +\sin\left(C_2\,\omega^2_{g'g}\right)\Big\{\sin(\phi)\,\Re\left[R^{S}_{g'}(\omega)+R^{AS}_{g'}(\omega)\right]-\cos(\phi)\,\Im\left[R^{S}_{g'}(\omega)-R^{AS}_{g'}(\omega)\right]\Big\}\Big]\end{gathered}}$$

As discussed in the Main Text, by experimentally tuning the PP chirp to the value $C'_2=\frac{2n\pi}{\omega^2_{g'g}}$ (with $n=0,\pm1,\pm2,\dots$), the measured signal is

$$\frac{S(\omega,T,C'_2)}{\operatorname{sgn}\Big(\frac{\partial\alpha}{\partial Q_{g'}}\Big)}\propto\cos(\phi')\,\Re\Big[R^{S}_{g'}(\omega)-R^{AS}_{g'}(\omega)\Big]+\sin(\phi')\,\Im\Big[R^{S}_{g'}(\omega)+R^{AS}_{g'}(\omega)\Big]$$

while for $C''_2=\left(n+\frac{1}{4}\right)\frac{2\pi}{\omega^2_{g'g}}$ it reads

$$\frac{S(\omega,T,C''_2)}{\operatorname{sgn}\Big(\frac{\partial\alpha}{\partial Q_{g'}}\Big)}\propto\sin(\phi'')\,\Re\Big[R^{S}_{g'}(\omega)+R^{AS}_{g'}(\omega)\Big]-\cos(\phi'')\,\Im\Big[R^{S}_{g'}(\omega)-R^{AS}_{g'}(\omega)\Big]$$

Hence, by tuning the time delay T and dechirping the experimental ISRS data[2], we can switch on and off the $\cos(\phi)$, $\sin(\phi)$ terms in the expressions of $S(\omega,T,C'_2)$ and $S(\omega,T,C''_2)$, separately accessing the real/imaginary part of the sum and the difference of complex REPs $R^{S}_{g'}(\omega)$ and $R^{AS}_{g'}(\omega)$. The measured signal corresponding to such values of the experimental parameters C_2 and $\tilde{T}$ is summarized in Table 1 of the Main Text.

In conclusion, we have shown that a proper tuning of the PP chirp C_2 and of the delay $\tilde{T}$, each in two selected configurations, ultimately leads to a full reconstruction of both the real and imaginary part of $R^{S}_{g'}(\omega)$ and $R^{AS}_{g'}(\omega)$.

SUPPLEMENTARY NOTE 2: STATIC ABSORPTION CORRECTION TO THE ISRS SIGNAL.

In this section, it will be discussed how the static absorption (SA) of the sample can be taken into account in the derivation of the measured signal $S(\omega,T)$.

Notably, in the considered experimental configuration, while the SA of the RP can be neglected in view of the off-resonant nature of the pump with respect to the sample absorbance profile (cf. Fig. 1(e) of the Main Text), the SA of the PP should be taken into account.

---

[2] indeed, the dechirped map corresponds to a map as a function of $\tilde{T}(\omega,T,C_2)=\frac{\phi}{\omega_{g'g}}$

The nonlinear wave equation [2, 5] for a beam propagating along the $\hat{z}$ direction inside a given medium reads

$$\frac{\partial^2 E(\mathbf{r},t)}{\partial z^2} - \frac{\epsilon}{c^2}\frac{\partial^2 E(\mathbf{r},t)}{\partial t^2} = \mu_0 \frac{\partial^2 P_{NL}(\mathbf{r},t)}{\partial t^2} \tag{S13}$$

with

$$\epsilon = \left(n(\omega) - i\,\frac{\hat{\alpha}(\omega)c}{2\,\omega}\right)^2$$

where $n(\omega)$ is the refractive index of the sample and $\hat{\alpha}(\omega)$ its absorption coefficient. We seek a solution with the form of quasi-monochromatic waves (i.e. monochromatic waves at frequency ω_0 multiplied by a slowly varying envelope):

$$E(\mathbf{r},t) = A(z,t)\, e^{i(\omega_0 t - k_0 z)}$$

$$P_{NL}(\mathbf{r},t) = p_{NL}(z,t)\, e^{i(\omega_0 t - k_0 z)}$$

where $k_0 = \frac{\omega_0}{c}\, n(\omega_0) = \frac{\omega_0}{c}\, n_0$. It is worth to stress that the absorption term is not present in the exponentials, since its effects will be included in the envelopes $A(z,t)$, $p_{NL}(z,t)$.

Under the Paraxial and the Slowly Varying Envelope Approximations (SVEA) [2, 5], the second derivative of $A(z,t)$ with respect to z and both the first and second time derivative of $A(z,t)$ and $p_{NL}(z,t)$ can be neglected, obtaining

$$\frac{\partial^2 E(z,t)}{\partial z^2} = -k_0^2\, A(z,t) e^{i(\omega_0 t - k_0 z)} - 2ik_0 \frac{\partial A(z,t)}{\partial z} e^{i(\omega_0 t - k_0 z)}$$

$$\frac{\partial^2 E(z,t)}{\partial t^2} = -\omega_0^2\, A(z,t)\, e^{i(\omega_0 t - k_0 z)}$$

$$\frac{\partial^2 P_{NL}(z,t)}{\partial t^2} = -\omega_0^2\, p_{NL}(z,t)\, e^{i(\omega_0 t - k_0 z)}$$

By replacing these expression in Eq.S13, and writing $\epsilon \approx n_0^2 - i n_0 \hat{\alpha}(\omega)\frac{c}{\omega_0}$ (neglecting the dispersion effects), we get

$$\left[- k_0^2 A(z,t) - 2ik_0 \frac{\partial A(z,t)}{\partial z} + \Big(\frac{n_0^2\omega_0^2}{c^2} - i\frac{\hat{\alpha}(\omega) n_0 \omega_0}{c}\Big) A(z,t)\right] e^{i(\omega_0 t - k_0\, z)} = -\mu_0\, \omega_0^2\, p_{NL}(z,t)\, e^{i(\omega_0 t - k_0 z)}$$

Hence, the nonlinear Helmholtz equation in the SVEA and in presence of the sample absorption reads

$$-2ik_0 \frac{\partial A(z,t)}{\partial z} = -\mu_0\, \omega_0^2\, p_{NL}(z,t) + i\frac{\alpha(\omega) n_0 \omega_0}{c} A(z,t)$$

and, by switching to the frequency domain via Fourier transformation,

$$-2ik_0 \frac{\partial A(z,\omega)}{\partial z} = -\mu_0\, \omega_0^2\, p_{NL}(z,\omega)\; + i\frac{\alpha(\omega) n_0 \omega_0}{c} A(z,\omega)$$

The decrease in the amplitude of the PP electric field due to SA can be explicitly taken into account by writing

$$A(z,\omega) = U_P(z,\omega)\, A_0\, e^{-\hat{\alpha}(\omega) z/2}$$

where A_0 is related to the peak-amplitude of the probe pulse before the sample, while $U_P(z,\omega)$ describes its spectral profile during the propagation along z. We get

$$-2ik_0 \left[\frac{\partial U_P(z,\omega)}{\partial z} - \frac{\hat{\alpha}(\omega)}{2} U_P(z,\omega)\right] A_0\, e^{-\hat{\alpha}(\omega) z/2} = -\mu_0\, \omega_0^2\, p_{NL}(z,\omega) + i\frac{\alpha(\omega) n_0 \omega_0}{c} U_P(z,\omega)\, A_0\, e^{-\hat{\alpha}(\omega) z/2}$$

which can be readily simplified to

$$\frac{\partial U_P(z,\omega)}{\partial z} A_0\, e^{-\hat{\alpha}(\omega) z/2} = -i\,\frac{\mu_0\, \omega_0^2}{2k_0}\, p_{NL}(z,\omega) \tag{S14}$$

The nonlinear polarization spectral profile $p_{NL}(z,\omega)$ is given by Eq.S8 for the A processes, and by an analogue expression for the B ones. They read

$$p_A(z,\omega) \propto -i\, VP^{RP}_{g'} \sum_k \mu_{g'\,e_k}\mu_{e_k\,g}\, \frac{U_P(z,\omega-\omega_{g'g})}{(\omega-\tilde{\omega}_{e_k g})} A_0\, e^{-\frac{\hat{\alpha}(\omega-\omega_{g'g})}{2} z} \tag{S15}$$

$$p_B(z,\omega) \propto i\, VP^{RP}_{g'} \sum_k \mu_{g\,e_k}\mu_{e_k\,g'}\, \frac{U_P(z,\omega+\omega_{g'g})}{(\omega-\tilde{\omega}_{e_k g'})} A_0\, e^{-\frac{\hat{\alpha}(\omega+\omega_{g'g})}{2} z} \tag{S16}$$

Notably, as anticipated above, the effect of the sample's absorption is included in the nonlinear polarization profile via the attenuation of the PP field $A(z,\omega\pm\omega_{g'g})$ which generates the $P^{(3)}$.

Firstly, we consider the **A class** of processes, which are related to the Feynman diagrams in Fig.1(a) of the Main Text. Eq.S14 for the PP spectral profile $U_P^A(z,\omega)$ modified by $p_A(z,\omega)$ can be recast via Eq.S15 as

$$\frac{\partial U_P^A(z,\omega)}{\partial z} \propto -\frac{\mu_0\,\omega_0^2}{2\,k_0}\, U_P^A(z,\omega-\omega_{g'g})\, e^{\frac{\hat{\alpha}(\omega)-\hat{\alpha}(\omega-\omega_{g'g})}{2} z} \tag{S17}$$

where the term $VP^{RP}_{g'} \sum_k \frac{\mu_{g' e_k}\mu_{e_k g}}{(\omega-\tilde{\omega}_{e_k g})}$ has been included in the proportionality factor as it does not depend on z. Notably, in view of the small intensity of the nonlinearly induced features, the modifications of the PP spectral profile along propagation are almost entirely due to $\hat{\alpha}(\omega)$. Hence, the $U_P^A(z,\omega-\omega_{g'g})$ dependence on z can be neglected in the r.h.s. of Eq.S17.

In absence of SA, namely if $\hat{\alpha}=0$, by integrating on the effective length L_e of the sample where the nonlinear process generating the signal takes place we get

$$\Delta U_P^A(\omega)\Big|_{\hat{\alpha}=0} = U_P^A(L_e,\omega)\Big|_{\hat{\alpha}=0} - U_P^A(0,\omega) \propto -\,U_P^A(0,\omega-\omega_{g'g})\; L_e$$

while if $\hat{\alpha}\neq 0$ the solution reads

$$\Delta U_P^A(\omega)\Big|_{\hat{\alpha}} = U_P^A(L_e,\omega)\Big|_{\hat{\alpha}} - U_P^A(0,\omega) \propto -\,U_P^A(0,\omega-\omega_{g'g})\; 2\,\frac{e^{\frac{\hat{\alpha}(\omega)-\hat{\alpha}(\omega-\omega_{g'g})}{2} L_e}-1}{\hat{\alpha}(\omega)-\hat{\alpha}(\omega-\omega_{g'g})}$$

Notably, the quantity $\Delta U_P^A(\omega)$ in the two preceding expressions represents the modification to the PP electric field spectral profile due to the nonlinear response in absence/presence of SA, respectively. It holds

$$\frac{\Delta U_P^A(\omega)\Big|_{\hat{\alpha}}}{\Delta U_P^A(\omega)\Big|_{\hat{\alpha}=0}} = 2\,\frac{e^{\frac{\hat{\alpha}(\omega)-\hat{\alpha}(\omega-\omega_{g'g})}{2} L_e}-1}{[\hat{\alpha}(\omega)-\hat{\alpha}(\omega-\omega_{g'g})]L_e}$$

The contribution related to the A processes in the heterodyne-detected ISRS signal can be expressed as[2]

$$S_A(L_e,\omega,T) \propto \Re\left[\frac{A_A^{(3)}(L_e,\omega,T)}{A_P(L_e,\omega)}\right]$$

where $A_A^{(3)}(L_e,\omega,T)$ is the (envelope of the) electric field generated by the relevant third-order processes, i.e.

$$A_A^{(3)}(L_e,\omega) = \Delta U_P^A(\omega)\, A_0\, e^{-\hat{\alpha}L_e/2}$$

while $A_P(\omega,L_e) = U_P(L_e,\omega)\, A_0\, e^{-\hat{\alpha}L_e/2}$. Hence,

$$\frac{S_A(L_e,\omega,T)\Big|_{\hat{\alpha}}}{S_A(L_e,\omega,T)\Big|_{\hat{\alpha}=0}} \approx \frac{\Delta U_P^A(\omega)\Big|_{\hat{\alpha}}}{\Delta U_P^A(\omega)\Big|_{\hat{\alpha}=0}} = 2\,\frac{e^{\frac{\hat{\alpha}(\omega)-\hat{\alpha}(\omega-\omega_{g'g})}{2} L_e}-1}{[\hat{\alpha}(\omega)-\hat{\alpha}(\omega-\omega_{g'g})]L_e}$$

The final expression for the **static absorption correction** to the ISRS signal related to the **A** processes is

$$\boxed{\frac{S_A(L_e,\omega,T)\Big|_{\hat{\alpha}}}{S_A(L_e,\omega,T)\Big|_{\hat{\alpha}=0}} = 2\,\frac{e^{\frac{\hat{\alpha}(\omega)-\hat{\alpha}(\omega-\omega_{g'g})}{2} L_e}-1}{[\hat{\alpha}(\omega)-\hat{\alpha}(\omega-\omega_{g'g})]L_e}} \tag{S18}$$

while the correction for the **B** ones can be analogously computed as

$$\boxed{\frac{S_B(L_e,\omega,T)\Big|_{\hat{\alpha}}}{S_B(L_e,\omega,T)\Big|_{\hat{\alpha}=0}} = 2\,\frac{e^{\frac{\hat{\alpha}(\omega)-\hat{\alpha}(\omega+\omega_{g'g})}{2}L_e}-1}{[\hat{\alpha}(\omega)-\hat{\alpha}(\omega+\omega_{g'g})]L_e}} \tag{S19}$$

SUPPLEMENTARY NOTE 3: THIRD ORDER DISPERSION EFFECTS ON THE ISRS SIGNAL.

When REPs are reconstructed over a broad range of the PP spectrum, it may be necessary to include higher order corrections to the dispersion terms besides the chirp C_2. In general, the probe field can be expanded as

$$E_P(\omega) = E_P^0(\omega)\; e^{i\sum_{n=2}^{\infty} C_n(\omega-\omega_P)^n}$$

In the following, the calculations presented in the Supplementary Note 1 are expanded by including the third order dispersion, so that $E_P(\omega) = E_P^0(\omega)\; e^{iC_2(\omega-\omega_P)^2}\; e^{iC_3(\omega-\omega_P)^3}$.

For small vibrational dephasing $\Gamma_{g'g}\approx 0$, the ISRS signal for the A diagrams in Eq.S9 can be rewritten as

$$S_A(\omega,T) \propto \mathrm{sgn}\Big(\frac{\partial\alpha}{\partial Q_{g'}}\Big)\cdot$$
$$\Re\left[\frac{E_P^0(\omega-\omega_{g'g})}{E_P^0(\omega)}\, e^{-i\omega_{g'g}T}\, e^{iC_2(\omega-\omega_P-\omega_{g'g})^2}\, e^{iC_3(\omega-\omega_P-\omega_{g'g})^3}\, e^{-iC_2(\omega-\omega_P)^2}\, e^{-iC_3(\omega-\omega_P)^3}\, R^S_{g'}(\omega)\right]$$

The terms with C_2, C_3 in the imaginary exponents of the preceding expression read

$$C_2(\omega-\omega_P-\omega_{g'g})^2 - C_2(\omega-\omega_P)^2 + C_3(\omega-\omega_P-\omega_{g'g})^3 - C_3(\omega-\omega_P)^3 =$$
$$= C_2\,\omega_{g'g}^2 - 2\,C_2(\omega-\omega_P)\,\omega_{g'g} - C_3\,\omega_{g'g}^3 - 3\,C_3(\omega-\omega_P)^2\omega_{g'g} + 3\,C_3(\omega-\omega_P)\,\omega_{g'g}^2$$

Hence, by defining the phase term for the A signal as

$$\boxed{\phi_A = \phi_A(\omega,T,C_2,C_3) = \omega_{g'g}T + (2\,C_2 - 3\,C_3\,\omega_{g'g})(\omega-\omega_P)\,\omega_{g'g} + 3\,C_3(\omega-\omega_P)^2\omega_{g'g}}$$

it holds

$$S_A(\omega,T) \propto \mathrm{sgn}\Big(\frac{\partial\alpha}{\partial Q_{g'}}\Big)\,\frac{E_P^0(\omega-\omega_{g'g})}{E_P^0(\omega)}\;\Re\left[e^{-i\,\phi_A}e^{i(C_2-C_3\,\omega_{g'g})\,\omega_{g'g}^2}\,R^S_{g'}(\omega)\right] \tag{S20}$$

Similarly, by including the third order dispersion term to Eq.S10, the $S_B(\omega,T)$ signal reads as

$$S_B(\omega,T) \propto \mathrm{sgn}\Big(\frac{\partial\alpha}{\partial Q_{g'}}\Big)\cdot$$
$$-\,\Re\left[\frac{E_P^0(\omega+\omega_{g'g})}{E_P^0(\omega)}\, e^{i\omega_{g'g}T}\, e^{iC_2(\omega-\omega_P+\omega_{g'g})^2}\; e^{iC_3(\omega-\omega_P+\omega_{g'g})^3}\, e^{-iC_2(\omega-\omega_P)^2}\, e^{-iC_3(\omega-\omega_P)^3}\, R^{AS}_{g'}(\omega)\right]$$

The phase term for the B signal is defined, in analogy to the previous one, as

$$\boxed{\phi_B = \phi_B(\omega,T,C_2,C_3) = -\omega_{g'g}T - (2C_2 + 3C_3\,\omega_{g'g})(\omega-\omega_P)\,\omega_{g'g} - 3C_3(\omega-\omega_P)^2\omega_{g'g}}$$

so that

$$S_B(\omega,T) \propto -\mathrm{sgn}\Big(\frac{\partial\alpha}{\partial Q_{g'}}\Big)\,\frac{E_P^0(\omega+\omega_{g'g})}{E_P^0(\omega)}\;\Re\left[e^{-i\,\phi_B}\;e^{i(C_2+C_3\,\omega_{g'g})\,\omega_{g'g}^2}\,R^{AS}_{g'}(\omega)\right] \tag{S21}$$

Being the RP off-resonant, in the PP impulsive limit ($E_P^0(\omega) = E_P^0$ in the region of interest) the ISRS signal measured for the mode g' is given by the sum of the two contributions in Eq.s S20, S21, namely

$$\boxed{S(\omega,T) \propto \mathrm{sgn}\Big(\frac{\partial\alpha}{\partial Q_{g'}}\Big)\,\Re\left[e^{-i\,\phi_A}\;e^{i(C_2-C_3\,\omega_{g'g})\,\omega_{g'g}^2}\;R^S_{g'}(\omega)\;-\;e^{-i\,\phi_B}\;e^{i(C_2+C_3\,\omega_{g'g})\,\omega_{g'g}^2}\;R^{AS}_{g'}(\omega)\right]} \tag{S22}$$

SUPPLEMENTARY NOTE 4: DUSCHINSKY MIXING OF THE NORMAL MODES.

The Duschinsky matrix [6], computed via TD-DFT, is shown in Supplementary Figure 1, revealing negligible coupling of each of the five modes investigated in this paper (red dashed lines in panels a-b) with respect to the others normal modes.

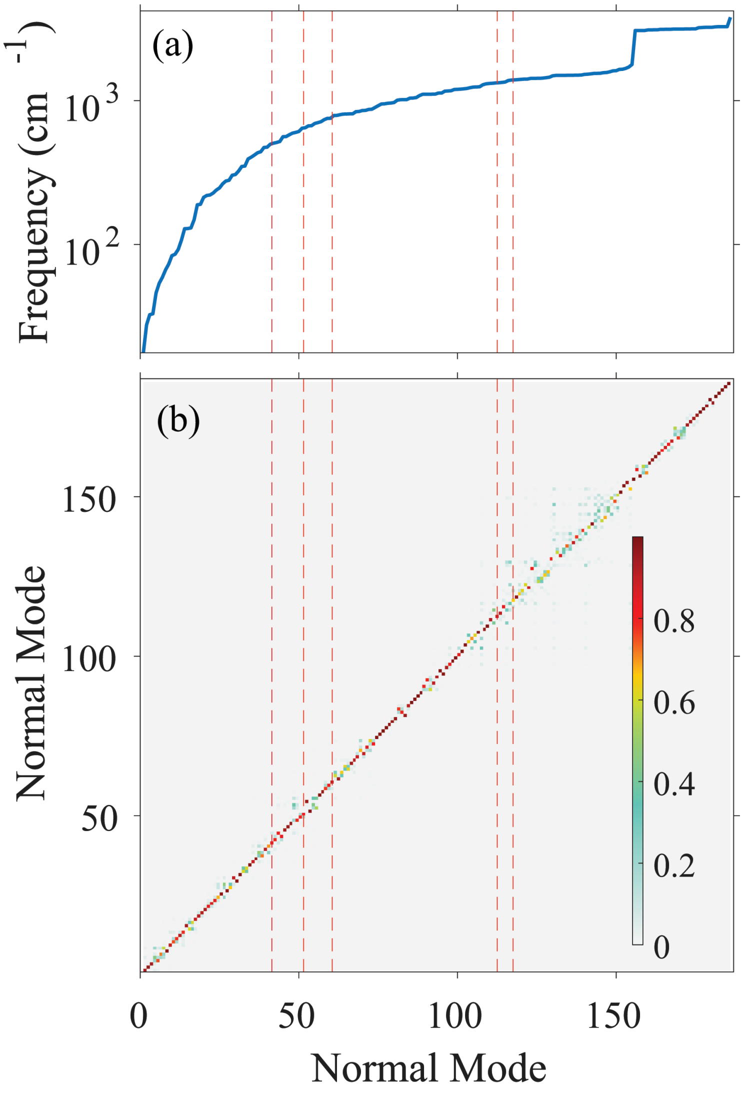

Supplementary Figure 1. Frequencies (panel a) and Duschinsky mixing (panel b) of the 186 normal modes of the Rhodamine B molecule, computed via TD-DFT (CAM-B3LYP functional and 6-311++g(2d,2p) basis set). The red dashed lines correspond to the five investigated modes at $490, 620, 735, 1280$ and 1360 cm^{-1} (experimental frequencies).

SUPPLEMENTARY NOTE 5: COMPARISON BETWEEN THE TRANSFORM THEORY AND THE LORENTZIAN LINESHAPE APPROXIMATION.

In this section the transform theory [7] is applied to derive an expression for the complex Raman excitation profiles, which can be conveniently implemented in numerical analyses. The definition of the REPs for the g' normal mode is reported in Eq.4 of the Main Text, which is rewritten in the following for convenience

$$R^S_{g'}(\omega) = \sum_{e_k} \frac{\mu_{g'\,e_k}\mu_{e_k\,g}}{\omega - \tilde{\omega}_{e_k g}}, \quad R^{AS}_{g'}(\omega) = \sum_{e_k} \frac{\mu_{g'\,e_k}\mu_{e_k\,g}}{\omega - \tilde{\omega}_{e_k g'}} = R^S_{g'}(\omega - \omega_{g'g}) \tag{S23}$$

Notably, the summation runs over the full vibrational manifold in the electronic excited-state, which includes a progression of eigenstates for each molecular normal mode. Indeed, a specific molecular state in an expanded notation can be expressed as

$$|e_k\rangle = |E\rangle\, |n_1\rangle^E_1\, |n_2\rangle^E_2 \cdots |n_N\rangle^E_N$$

being the direct product of the electronic state ($|G\rangle$ or $|E\rangle$ for the ground or the excited one, respectively) with the $|n_j\rangle^{G/E}$ vibrational state related to the j-th normal mode ($j = 1, 2, ..., N$, where is the number of normal modes of the molecule under investigation - $N = 186$ in the case studied here-, while $n_j = 0, 1, 2, ...$). Hence, by omitting hereafter the factor $\left|\langle G|E\rangle\right|^2$ for simplicity,

$$\begin{aligned}
\mu_{g'\,e_k}\mu_{e_k\,g} &= \Big({}^G_1\langle 0| \cdots {}^G_{g'}\langle 1| \cdots {}^G_N\langle 0| \Big)\Big(|n_1\rangle^E_1\, |n_2\rangle^E_2 \cdots |n_N\rangle^E_N \Big) \\
&\quad \Big({}^E_1\langle n_1|\; {}^E_2\langle n_2| \cdots {}^E_N\langle n_N| \Big)\Big(|0\rangle^G_1 \cdots |0\rangle^G_{g'} \cdots |0\rangle^G_N \Big) = \\
&= \left|{}^G_1\langle 0|n_1\rangle^E_1\right|^2 \cdots {}^G_{g'}\langle 1|n_{g'}\rangle^E_{g'}\; {}^E_{g'}\langle n_{g'}|0\rangle^G_{g'} \cdots \left|{}^G_N\langle 0|n_N\rangle^E_N\right|^2
\end{aligned}$$

As a first approximation, a closed-form expression for the REPs can be found by restricting the vibronic transitions to the g' mode only, i.e. by assuming $n_j = 0$ for any j different from g'. This is equivalent to assume $\left|{}^G_j\langle 0|0\rangle^E_j\right|^2 \gg \left|{}^G_j\langle 0|n_j\rangle^E_j\right|^2$ for any $n_j \geq 1$. In this case, Eq.S23 reads

$$R^S_{g'}(\omega) = \sum_{n_{g'}=0}^{3} \frac{{}^G_{g'}\langle 1|n_{g'}\rangle^E_{g'}\; {}^E_{g'}\langle n_{g'}|0\rangle^G_{g'}}{\omega - \omega_{e_0 g} - n_{g'}\,\omega_{g'g} + i\Gamma_{eg}}\;, \qquad R^{AS}_{g'}(\omega) = \sum_{n_{g'}=0}^{3} \frac{{}^G_{g'}\langle 1|n_{g'}\rangle^E_{g'}\; {}^E_{g'}\langle n_{g'}|0\rangle^G_{g'}}{\omega - \omega_{e_0 g} - (n_{g'} - 1)\,\omega_{g'g} + i\Gamma_{eg}} \tag{S24}$$

where, as the numerators become negligible for $n_{g'} \gg 1$ for small (<1) displacements, the summation indexes have been safely restricted to $n_{g'} = 0, 1, 2, 3$.

Notably, each of the four contributions to the REPs is shifted by one vibrational quantum $\omega_{g'g}$ with respect to the preceding one. As shown in Supplementary Figure 2 (a)-(b), respectively, their real parts are characterized by the sum of dispersive (shifted) profiles, while the imaginary ones by the sum of (shifted) positive or negative Lorentzian profiles. This ultimately leads to complex REPs critically dependent on the electronic dephasing Γ_{eg} and on the excited-state displacement d.

A more accurate expression for the REPs can be computed[7, 8] exploiting a Kramers-Kronig transform method in order to compute the linear Franck-Condon coupling strengths of low-frequency vibrational modes in the low coupling limit. Such a model, at odd with the one presented above, involves the absorption profile of the molecule, hence taking into account the full vibrational spectrum. In such a formalism, the expressions for $R^S_{g'}(\omega)$ and $R^{AS}_{g'}(\omega)$ read as

$$\begin{cases}
R^S_{g'}(\omega) = \sum\limits_{n_{g'}=0}^{3} {}^G_{g'}\langle 1|n_{g'}\rangle^E_{g'}\; {}^E_{g'}\langle n_{g'}|0\rangle^G_{g'}\;\; \Phi(\omega - n_{g'}\,\omega_{g'g}) \\
R^{AS}_{g'}(\omega) = \sum\limits_{n_{g'}=0}^{3} {}^G_{g'}\langle 1|n_{g'}\rangle^E_{g'}\; {}^E_{g'}\langle n_{g'}|0\rangle^G_{g'}\;\; \Phi(\omega + (1 - n_{g'})\,\omega_{g'g})
\end{cases} \tag{S25}$$

where

$$\Phi(\omega) = \frac{1}{\pi} \fint d\omega' \frac{\tilde{A}(\omega')}{\omega'(\omega' - \omega)} \; + \; i\,\frac{\tilde{A}(\omega)}{\omega} \tag{S26}$$

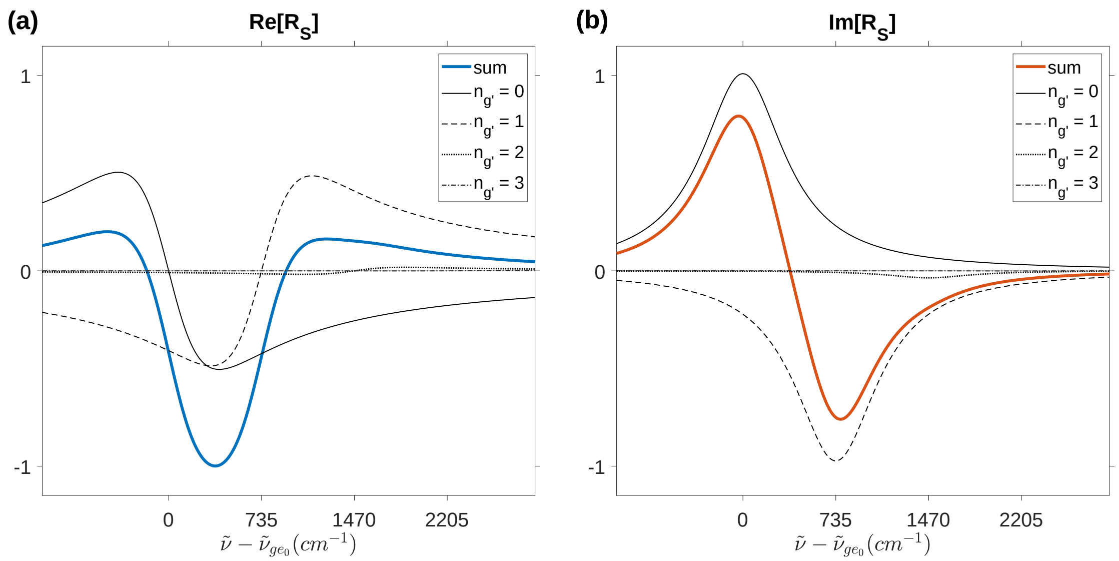

Supplementary Figure 2. Real (panel a) and Imaginary (panel b) parts of $R^S_{g'}(\omega)$ in the 1D approximation. $R^S_{g'}(\omega)$, whose absolute value is normalized, is computed for $\tilde{\nu}_{g'g} = 735\,cm^{-1}$, $d = -0.19$ and $\Gamma_{eg} = 400\,cm^{-1}$. The four contributions corresponding to $n_{g'g} = 0, 1, 2, 3$ in Eq.S24 are reported with black curves, as indicated in the legends.

In the definition of $\Phi(\omega)$, $⨍$ indicates a principal value integral, while $\tilde{A}(\omega)$ is the static absorption cross section related to vibronic transitions involving an excited-state (N-1) dimensional vibrational manifold, from which the g' normal mode is excluded. For small molecular displacements $\tilde{A}(\omega)$ can be approximated with the sample's absorbance profile.

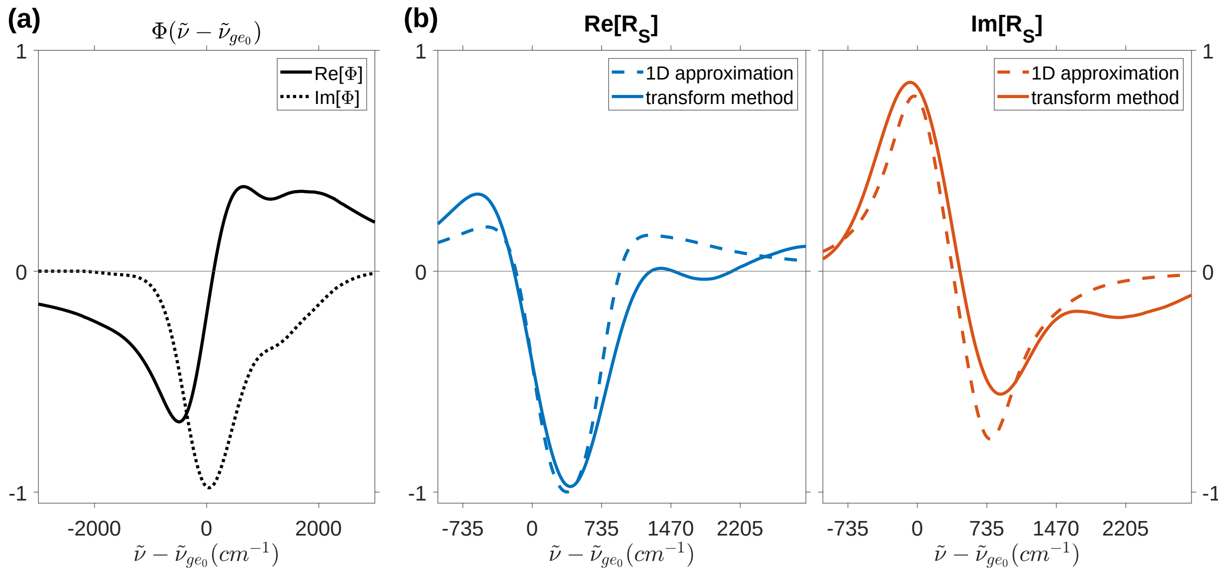

Supplementary Figure 3. (a): Complex profiles of $\Phi(\omega)$, reconstructed via Eq.S26 from the measured absorbance of the sample. (b): Comparison between the real and imaginary part of $R^S_{g'}(\omega)$ computed in the 1D approximation as in Supplementary Figure 2 (dashed curves) and via the transform method (solid ones) ($\tilde{\nu}_{g'g} = 735\,cm^{-1}$, $d = -0.19$, $\tilde{\nu}_{g\,e0} = 552\,nm$). Both $\Phi(\omega)$ and $R^S_{g'}(\omega)$ are normalized to the maximum of their absolute value.

The real and imaginary parts of $\Phi(\omega)$ are shown in Supplementary Figure 3(a), while the profiles of $R^S_{g'}(\omega)$ reconstructed with the transform method (Eq.S25) in Supplementary Figure 3(b) as solid curves. The dashed ones represent the corresponding REPs in the 1D approximation (Eq.S24), for comparison.

In Supplementary Figure 4, the dependence of the REPs on the molecular displacements, originating from the overlap of the vibrational eigenfunctions $\langle 1|n_{g'}\rangle^E_{g'}\ {}^E_{g'}\langle n_{g'}|0\rangle^G_{g'}$ and $\langle 1|n_{g'}\rangle^E_{g'}\ {}^E_{g'}\langle n_{g'}|0\rangle^G_{g'}$ terms in Eqs. S25, is shown. It is

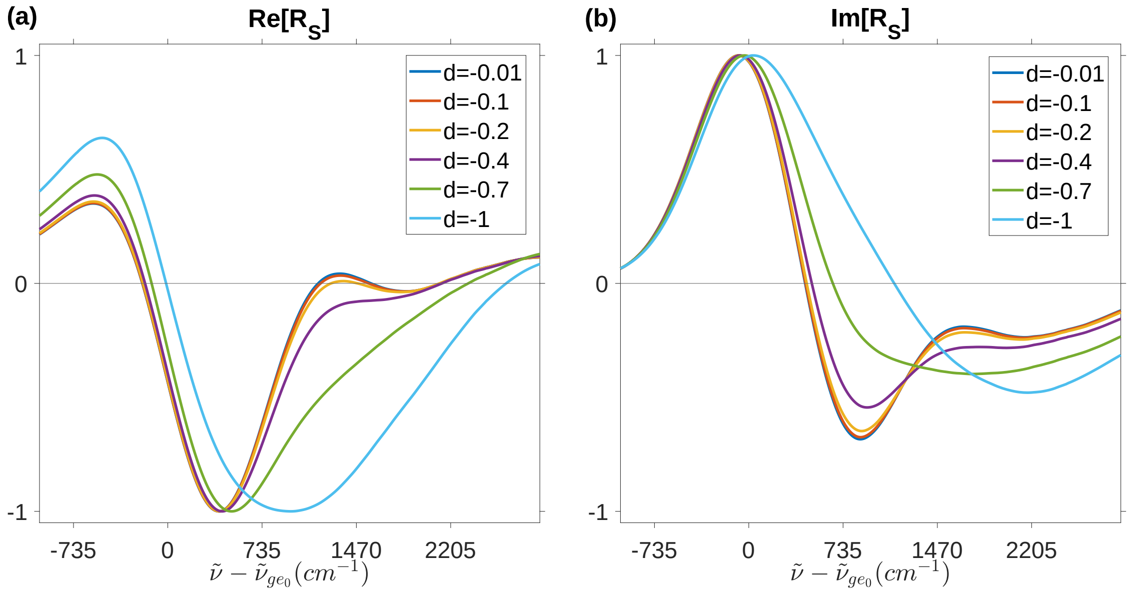

Supplementary Figure 4. Normalized real (panel a) and imaginary (panel b) parts of $R^S_{g'}(\omega)$ computed via the transform method for different values of the adimensional molecular displacement $d = Q\sqrt{\frac{m\,\omega_{g'g}}{2\hbar}}$ ($\tilde{\nu}_{g'g} = 735\,cm^{-1}$, $\tilde{\nu}_{g\,e0} = 552\,nm$).

worth to stress that the dependence of the normalized REP reported in Supplementary Figure 4 is highly sensitive to the magnitude of the displacement, which hence can be accessed directly from the spectral dependence of the REPs. For small amplitudes of the ES displacement, such dependence is less pronounced: under such regime, the relative amplitudes of the displacements along the different normal modes can be obtained from the relative intensities of the ISRS signals, and the absolute displacements can be retrieved by fitting the absorption profile.

SUPPLEMENTARY NOTE 6: TIME-DOMAIN FITS OF THE RECORDED ISRS MAPS.

Beside extracting the complex REPs, Eqs. 6, S18, S19, S22 and S25 can be also exploited to directly model the ISRS signal in the time-domain. This is illustrated in Supplementary Figure 5, where the experimental maps corresponding to the five investigated normal modes are reported in the first rows for selected probe chirps (namely, $C_2 = -4$ and $30\,fs^2$) and compared with the globally fitted ones (second rows). In order to calculate the vibrational preparation function (Eq. 3 of the Main Text), the absolute values of the molecular polarizability derivative $\left|\frac{\partial\alpha}{\partial Q_j}\right|$ have been taken from off-resonant Raman, as detailed in the Supplementary Note 7. The (normalized) values related for the five investigated normal modes Q_j at 490, 620, 735, 1280, 1360 cm^{-1} are 0.32, 0.64 , 0.47 , 0.68 , 1.00, respectively. Additionally, the 620 cm^{-1} displacement modulus has been fixed to fit the absorption spectrum, as discussed in the Main Text. The investigated vibrational modes have been isolated from the recorded ISRS signal, via a sinusoidal fit (see Fig.3 of the Main Text). The last rows of Supplementary Figure 5 show the difference between the two upper colormaps, i.e. the difference between the data and the model. Vertically stacked maps correspond to a single normal mode and share the same colorbar, reported in the bottom. We note that such an approach, taking advantage of the sensitivity of the recorded data to the femtosecond vibrational periods, can be exploited for a fine calibration of the experimental parameters (C_2, C_3 and T_0).

SUPPLEMENTARY NOTE 7: FREQUENCY-DOMAIN STIMULATED RAMAN SCATTERING MEASUREMENTS

As anticipated in the previous section, the magnitudes of the molecular polarizability derivatives $\left|\frac{\partial\alpha}{\partial Q_j}\right|$, used to model the ISRS response for the five investigated modes, have been fixed from conventional off-resonant frequency-domain Raman measurements. Specifically, the Stimulated Raman Scattering (SRS) [9, 10] response of Rhodamine B (RhB) dissolved in methanol has been acquired, using an off-resonant narrowband/picosecond Raman pump (central wavelength tuned at $\lambda_R \approx 638$ nm, and $\tau_R \approx 2$ ps time duration) and a broadband probe pulse. The recorded

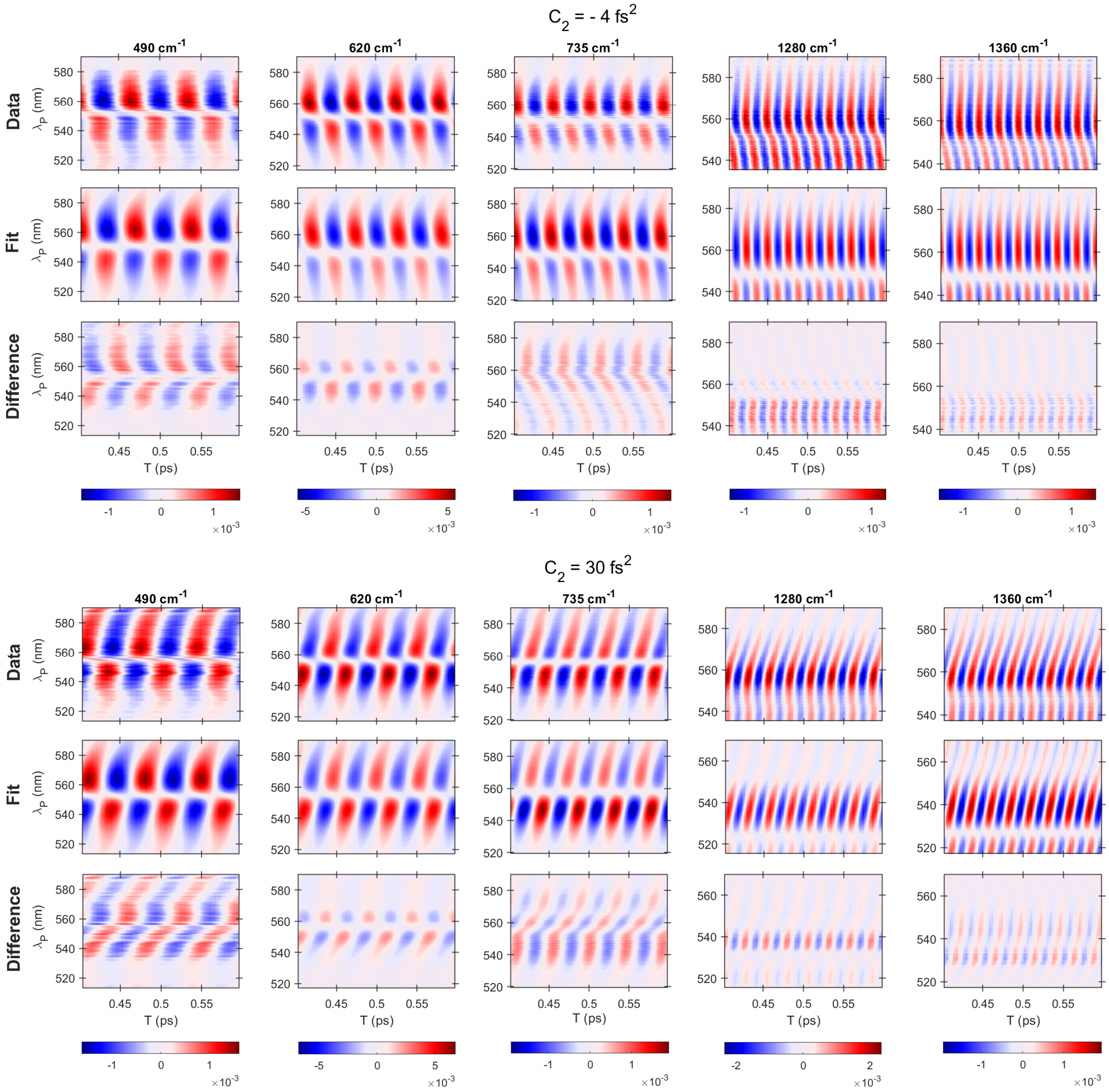

Supplementary Figure 5. Time-domain global fit of the ISRS signal. In each of the two experimental configurations ($C = -4 fs^2$ upper panel, $C = 30\ fs^2$ lower one), the contributions of the main five vibrational modes have been isolated in separate maps, reported in the first rows. The corresponding globally fitted maps are reported in the second rows, while the differences between the experimental data and the model are shown in the third ones for comparison.

stimulated Raman bands, which are proportional to the square modulus of the the molecular polarizability derivatives $\left|\frac{\partial \alpha}{\partial Q_j}\right|^2$, have been monitored in the red side of the spectrum, by looking at the Raman gains of the different PP spectral components. A representative SRS measurement of Rhodamine B (RhB) dissolved in methanol is shown in Supplementary Figure 6, in the $[315, 1407]$ cm^{-1} spectral range. It is worth to note that it also includes a strong Raman mode at 1035 cm^{-1} from the solvent, as well as other minor vibrational bands.

A magnification around the five normal modes investigated in this work (at $\sim$ 490, 620, 735, 1280 and 1360 cm^{-1}) is presented in Supplementary Figure 7: the five colormaps report the SRS signal as a function of the Raman shift ($\tilde{\nu} - \tilde{\nu}_R$) and of the pump-probe delay ΔT between the Raman and probe pulses. SRS profiles at selected temporal delays ΔT, i.e. horizontal slices of the colormaps, are reported in the corresponding upper panels [11].

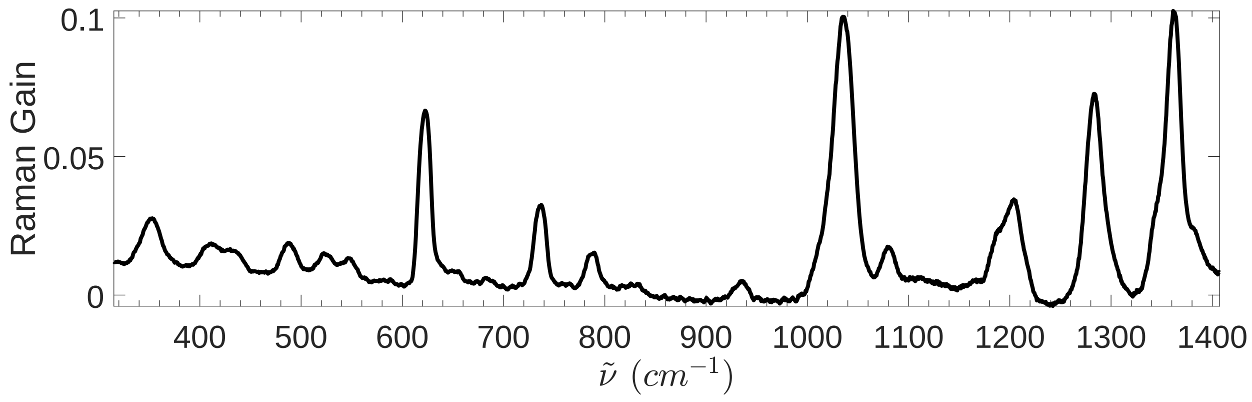

Supplementary Figure 6. Off-resonant stimulated Raman spectra of Rhodamine B dissolved in methanol.

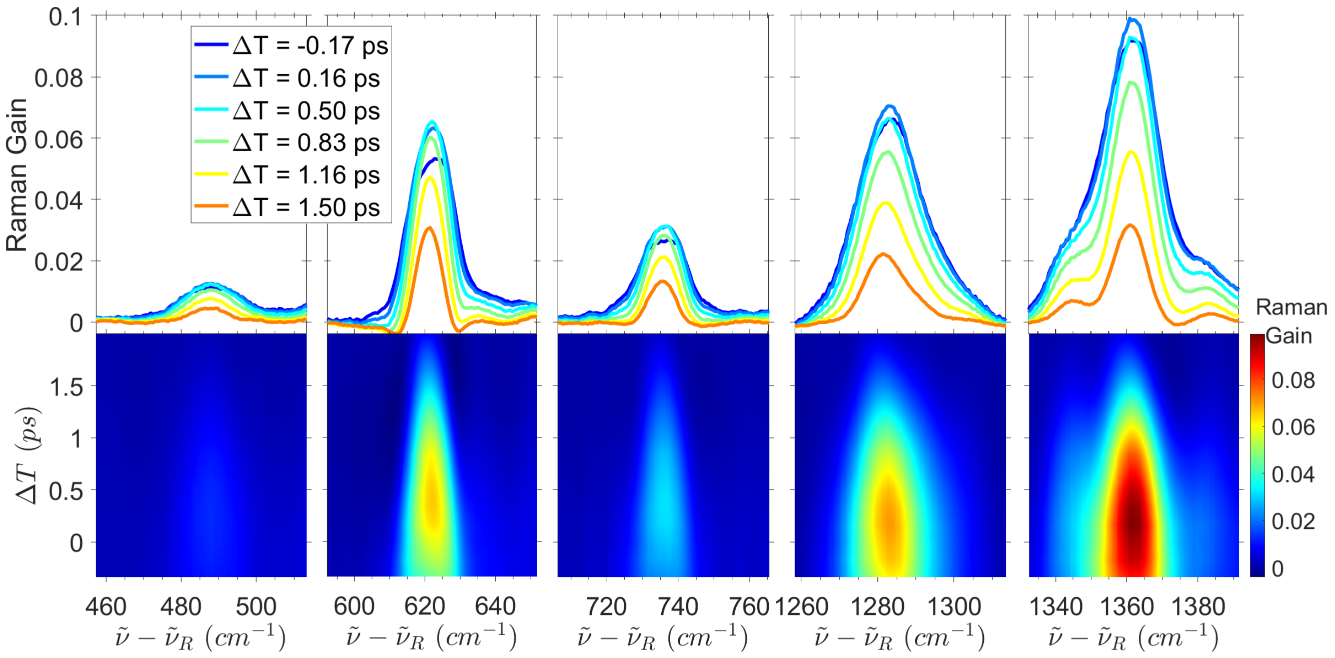

Supplementary Figure 7. Stimulated Raman Scattering response of RhB for the five normal Raman bands investigated in this work and recorded with a narrowband off-resonant Raman pump (λ_R = 638 nm). The colormaps, which are restricted to the spectral regions of interest, show the SRS signal as a function of the pump-probe delay ΔT between the Raman and probe pulses and of the Raman shift $\tilde{\nu} - \tilde{\nu}_R$. In the upper panels slices of SRS spectra are reported for selected ΔT time-delay values.

SUPPLEMENTARY NOTE 8: DFT CALCULATIONS.

Density functional theory (DFT) and TD-DFT calculations have been performed with CAM-B3LYP [12] functional and 6-311++g(2d,2p) basis set, by using the Gaussian 16 software package [13]. The normal modes eigenvectors calculated in the ground state have been exploited to generate multiple displaced geometries along the different normal modes under consideration, which have then been exploited to calculate by DFT the energy in the excited electronic state and the molecular polarizability. In Supplementary Figure 8, we report the extracted PES along the different normal mode projections and the corresponding polarizability. The circles indicate the energies obtained from DFT and TD-DFT calculations, while the colored parabolas are parabolic fits. For the ES, fits have been extracted either fixing the eigenfrequencies to the ground state values (continuous red lines) and setting them as a fitting parameter (dashed orange lines).

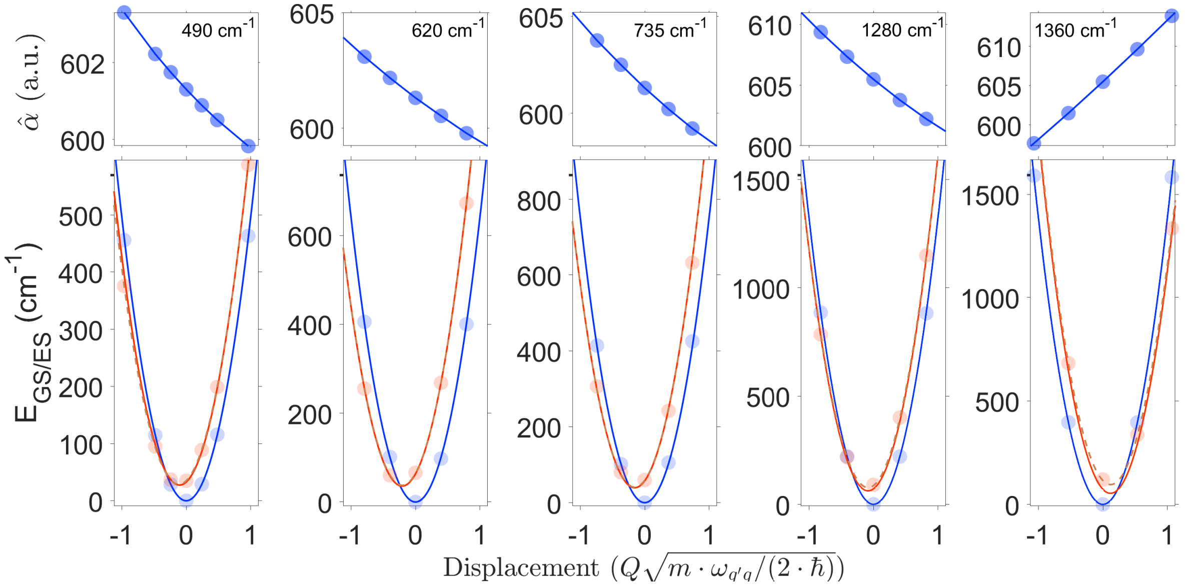

Supplementary Figure 8. One dimensional projections of the potential energy surfaces along the different normal modes investigated are reported as a function of the nuclear displacement. Calculations, performed using CAM-B3LYP functional and 6-311++G(2d,2p) basis set, are reported for the ground (blue lines) and excited (red) electronic states. The circles indicate the energies obtained from DFT and TD-DFT calculations, while the colored lines are a parabolic fit. For the fit of the excited state projections, the eigenfrequencies have been fixed to the GS values (red line) or set a free parameters (orange dashed line), obtaining similar results. In the top panels the ground state polarizabilities are reported as a function of the nuclear displacement along the five normal modes, defining the choice of the reference frame performed in the paper (cf. Fig.1 of the Main Text)

SUPPLEMENTARY REFERENCES

[1] Condon, E. U. The Franck-Condon Principle and Related Topics. *Am. J. Phys.* **1947**, *15*, 365–374.

[2] Mukamel, S. *Principles of Nonlinear Spectroscopy*; Oxford University Press: New York, 1995.

[3] Mukamel, S. Controlling multidimensional off-resonant-Raman and infrared vibrational spectroscopy by finite pulse band shapes. *The Journal of chemical physics* **2009**, *130*, 054110.

[4] Tanimura, Y.; Mukamel, S. Two-dimensional femtosecond vibrational spectroscopy of liquids. *J. Chem. Phys* **1993**, *99*, 9496–9511.

[5] Agrawal, G. *Nonlinear Fiber Optics*; Academic Press, 2013.

[6] Duschinsky, F. The importance of the electron spectrum in multi atomic molecules. Concerning the Franck-Condon principle. *Acta Physicochim. URSS* **1937**, *7*, 551–566.

[7] Stallard, B. R.; Champion, P. M.; Callis, P. R.; Albrecht, A. C. Advances in calculating Raman excitation profiles by means of the transform theory. *J. Chem. Phys.* **1983**, *78*, 712–722.

[8] Champion, P. M.; Albrecht, A. C. Resonance Raman Scattering: The Multimode Problem and Transform Methods. *Annu. Rev. Phys. Chem.* **1982**, *33*, 353–376.

[9] Kukura, P.; McCamant, D. W.; Mathies, R. A. Femtosecond stimulated Raman spectroscopy. *Annu. Rev. Phys. Chem.* **2007**, *58*, 461–488.

[10] Batignani, G.; Fumero, G.; Mai, E.; Martinati, M.; Scopigno, T. Stimulated Raman lineshapes in the large lightmatter interaction limit. *Opt. Mater. X* **2022**, *13*, 100134.

[11] Yoon, S.; McCamant, D. W.; Kukura, P.; Mathies, R. A.; Zhang, D.; Lee, S. Dependence of line shapes in femtosecond broadband stimulated Raman spectroscopy on pump-probe time delay. *J. Chem. Phys* **2005**, *122*, 024505.

[12] Yanai, T.; Tew, D. P.; Handy, N. C. A new hybrid exchange–correlation functional using the Coulomb-attenuating method (CAM-B3LYP). *Chem. Phys. Lett.* **2004**, *393*, 51–57.

[13] Frisch, M. J.; Trucks, G. W.; Schlegel, H. B.; Scuseria, G. E.; Robb, M. A.; Cheeseman, J. R.; Scalmani, G.; Barone, V.; Petersson, G. A.; Nakatsuji, H. et al. Gaussian 16 Revision C.01. 2016; Gaussian Inc. Wallingford CT.
